# Single-cell transcriptome atlas in C57BL/6 mice encodes morphological phenotypes in the aging kidneys

**DOI:** 10.1186/s12882-024-03514-0

**Published:** 2024-04-19

**Authors:** Shanzhi Yang, Peimin Liu, Yan Zhang, Haosen Xu, Jinyi Lan, Huan Jiang, Guoxiang Jin, Xiaoyan Bai

**Affiliations:** 1 Department of Nephrology, Guangdong Provincial People’s Hospital (Guangdong Academy of Medical Sciences), Southern Medical University, Guangzhou, China; 2 Guangdong-Hong Kong Joint Laboratory on Immunological and Genetic Kidney Diseases, Guangzhou, China

**Keywords:** Kidney, Histopathology, Single-cell transcriptomics, C57BL/6 mice, Podocyte, GBM, Knob-like structures

## Abstract

C57BL/6 mice are frequently utilized as murine models with the desired genetic background for altertion in multiple research contexts. So far, there is still a lack of comprehensive kidney morphology and single-cell transcriptome atlas at all stages of growth of C57BL/6 mice. To provide an interactive set of reference standards for the scientific community, we performed the current study to investigate the kidney’s development throughout the capillary-loop stage until senescence. Eight groups, with five to six mice each, represented embryonic stage (embryos 18.5 days), suckling period (1 day after birth), juvenile stage (1 month old), adulthood (containing 3 months old, 6 months old and 10 months old), reproductive senescence stage (20 months old), and post-senescence stage (30 months old), respectively. With age, the thickness of the glomerular basement membrane (GBM) was increased. Notably, GBM knobs appeared at three months and became frequent with age. Using single-cell transcriptome data, we evaluated how various biological process appear in particular cell types and investigated the potential mechanism of formation of GBM konbs. In conclusion, having access to detailed kidney morphology and single-cell transcriptome maps from C57BL/6 mice at various developmental stages of C57BL/6 mice would be a novel and major resource for biological research and testing of prospective therapeutic approaches.

## Introduction

The use of animal models in disease pathogenesis research provides numerous benefits. The C57BL/6 mouse strain, which exhibits inbreeding, has been extensively used as a foundational strain for the development of genetic animal models. Despite their high reproductive capacity, long lifespans, and low tumor susceptibility, these mice demonstrate a notable resistance to the progression of renal damage in experimental models of kidney disorders. To overcome this obstacle, researchers have used multiple approaches to enhance the successful modelling rate, hence improving the investigation of disease pathogenesis [1, 2]. In general, the evaluation process involves the examination of the morphological phenotype and histopathological elements of the kidney. Yet it is essential to establish the assessment criteria in animal studies by assessing the normal morphology and single-cell transcriptome atlas under physiological conditions. To this end, it is of great need to create comprehensive atlas of kidney histopathology at various growth periods of C57BL/6 mice as a valuable reference for researchers to obtain wider knowledge about this subject.

Single-cell sequencing (scRNA-seq) technology has contributed to our knowledge of the disease via a transcriptomic approach. The construction of a transcriptome map of human kidney cells would facilitate the investigation of renal cell biology and provide insight of the intricate connections between cell types and diseases [3]. In the past few years, numerous studies of research have provided insight into novel genes and pathways participating in the pathogenesis and progression of renal disorders [4–6]. Profiling different glomerular cells has been characterized to offer individual datasets for stratifying disease stages and dissecting glomerular function at the single-cell level [7, 8]. In streptozotocin-induced type 1 diabetic mouse models, the mRNA profile of glomerular cells has been evaluated to indicate genes involved in the podocyte injury [9]. Profiling of various immune cells revealed the possible role of different subtypes of macrophages in different stages of diabetic nephropathy using scRNA-seq technology [10]. The heterogeneity of endothelial cells (ECs) across and within tissues in C57BL/6 mice has also been inventoried using the scRNA-seq technology [11]. Nevertheless, single-cell sequencing enables the identification of cellular function in a detailed manner.

To establish a thorough atlas of kidney histopathology and single-cell transcriptome analysis in C57BL/6 mice, this work was set up to investigate the kidney from the capillary-loop stage of development to senescence at 30 months of age.

## Materials and methods

### Ethics statement

The research procedure follows the principles outlined in the Care and Use of Laboratory Animals published by the US National Institutes of Health (NIH Publication No. 85 − 23, modified 1996). Additionally, it has received approval from the Animal Ethics Committee at Guangdong Provincial People’s Hospital in Guangzhou, China.

### Animals

Eight groups of C57BL/6 mice, each with five to six animals (male and female are evenly proportioned), were assessed at different stages: embryo (18.5 days), new-born (1 day), adolescent (1 month), young (3 months), adulthood (6 months), middle-aged (10 months), old (20 months), and senescent (30 months) [12].

### Blood and urine chemistry

Blood samples were obtained at each stage using the retro-orbital method under isoflurane anesthesia. Additionally, body weight (BW) and kidney weight (KW) was measured and documented. Urine sample was collected using the reflex urination strategy at 9:00–10:00 AM. Blood glucose, serum blood urea nitrogen (BUN), and serum and urine creatinine levels were measured using a Beckman Coulter AU480 Chemistry Analyzer (Beckman). The evaluation of urine albumin was conducted using a mouse-specific albumin ELISA kit (E99-134, Bethyl Laboratories, Montgomery, TX). The results were reported as the urine albumin to creatinine ratio (UACR). Every experiment was carried out three times.

### Tissue collection and animal euthanasia

Pentobarbital sodium (P3761, 30 mg/kg, Sigma-Aldrich, St. Louis, MO) was injected intraperitoneally into eight groups of mice to anesthetize them. Their body weight was then measured. The left kidneys were infused with 0.1 M PBS (pH = 7.4) at 80 to 100 mmHg for two minutes through the abdominal artery to remove circulating blood. Kidney weight (KW) was measured as the left kidneys were quickly removed.

### Histology analysis

Tissue slices were fixed in 10% formalin for 24 h, rinsed in buffer, dehydrated, and embedded in paraffin for light microscopy and histological examination. 2 μm thick paraffin sections were cut, stained with hematoxylin and eosin (H&E) for examination of cell structure, periodic acid-Shiff (PAS) and periodic acid-silver methenamine (PASM) to detect alterations in basement membrane architecture and glycogen deposition. Masson’s trichrome staining (MTS) and Picric acid-Sirius red stain were used to demonstrate the deposition of collagen matrix. At least 20 randomly selected kidney sections from each mouse were evaluated for structural alterations. Two masked independent investigators analyzed twenty glomeruli in each of the twenty fields per section, using a 100 objective oil immersion lens.

### Transmission electron microscopy

Several 1-mm cubes were cut from the cortex of left kidneys, fixed in 2.5% glutaraldehyde for no less than four hours, rinsed with cacodylate buffer, postfixed in 1% osmium tetroxide, and block-stained in uranyl acetate before being embedded in Poly/Bed812 resin (Polysciences, Inc., Warrington, PA). Ultrathin sections were procured from a minimum of three glomeruli that were randomly chosen from each animal. These sections were then examined by staining using uranyl acetate and lead citrate. Digital micrographs of the glomeruli from each group of mice were captured using a Hitachi 7700 transmission electron microscope (Tokyo) at magnifications of 2000 and 6000.

### Morphometric analysis of the kidney

On PAS-stained kidney tissue sections from 30 glomeruli from each group of mice, the degree of mesangial expansion was assessed to determine the degree of glomerular injury. Assessment of the mesangial and glomerular cross-sectional areas was performed using Image Pro Plus software (Media Cybernetics, Bethesda, MD) [13]. Evaluation of GBM thickness and podocyte foot process width were performed on five glomeruli with electron microscope [14, 15]. Using an automated analyzer in the transmission electron microscope, the thickness of the GBM was measured (Hitachi 7700, Hitachi High Technologies Corporation, Japan). By calculating the proportion of the effaced foot process per length of the glomerular basement membrane, podocyte foot process effacement was determined. In the analysis, the mean value was noted and used.

### Single cell separation

Tissues were washed three times with PBS, cut into blocks of 2 mm and placed on DMEM-1640 medium (Gibco, Gaithersburg MD, USA). Using buffer containing collagenase IV (1 mg/ml; Thermo Fisher), pronase E (1 mg/ml), and DNase I (50 U/ml), the tissues were digested at 37 °C for 30 min. Cells were filtered through a 70 μm cell strainer and centrifuged at 400×g for 5 min. Take a small amount of single-cell suspension, add 0.4% trypan blue staining solution of the same volume, count the cells with Countess II Automated Cell Counter, and adjust the concentration of living cells to the ideal range (1000 to 2000 cells/µl).

### Library construction and scRNA-seq

The gel beads containing barcode information combine with a mixture of cells and enzymes,then enter the reservoir to be separated by oil to form GEMs (GelBeads-In-Emulsions). After that, the gel beads dissolved and released the capture sequence containing the Barcode sequence, reverse transcribed the cDNA fragment, and labeled the sample. Break up the gel beads and oil droplets then use cDNA as a template for PCR amplification. All the products of GEMs are mixed to construct a standard sequencing library. The cDNA was cut into 200 ∼ 300 bp fragments, then the second-generation sequencing library was constructed. Finally, the DNA library was amplified by PCR. The high-throughput sequencing of the library was carried out by using the PE150 sequencing mode of the Illumina sequencing platform. Quality control of sequencing was performed using cellranger.

### Bioinformatics analysis

The expression matrix was transferred to Scanpy for subsequent analysis, and a set of cells were clustered according to both cell types and cell subgroups by singleR and Seurat. Gene differential expression analysis was performed separately for different classes of cell populations using a rank sum test to screen subgroups for up-regulated expression genes. Gene Ontology (GO; http://geneontology.org/) and Reactome pathway enrichment analyses were used to perform functional enrichment analyses using Homo sapiens as the genetic model.

### Immunofluorescence

Immunofluorescence microscopy was performed on snap-frozen kidney tissues to rule out immunological complex diseases involving IgG, C3, IgA, IgM, and C1q. Kidney tissues embedded in paraffin and formalin-fixed were used to detect the expression profile of specific kidney-related markers. Mouse monoclonal synaptopodin (1:200) (sc-515842, Santa Cruz Biotechnology, Dallas, TX, USA) and rabbit polyclonal Wilm’s Tumor 1 (WT1) antibody (1:100) (ab89901, Abcam, Cambridge, MA, USA) were the primary antibodies utilized. The FV1000-IX81 confocal laser scanning microscope (Olympus, Germany) was used to capture the images. Primary antibodies were replaced with PBS as the negative control.

### Statistical analysis

The previously data are shown as mean ± SD. One-Way ANOVA and Two-Way ANOVA followed by the Tukey’s post hoc test was utilized to determine the statistical significance between groups. All statistical analyses were carried out using version 25.0 of SPSS and GraphPad Prism 10.0. A significance level of 0.05 was established.

## Results

### Biological parameters at various stages

**Fig. 1:**
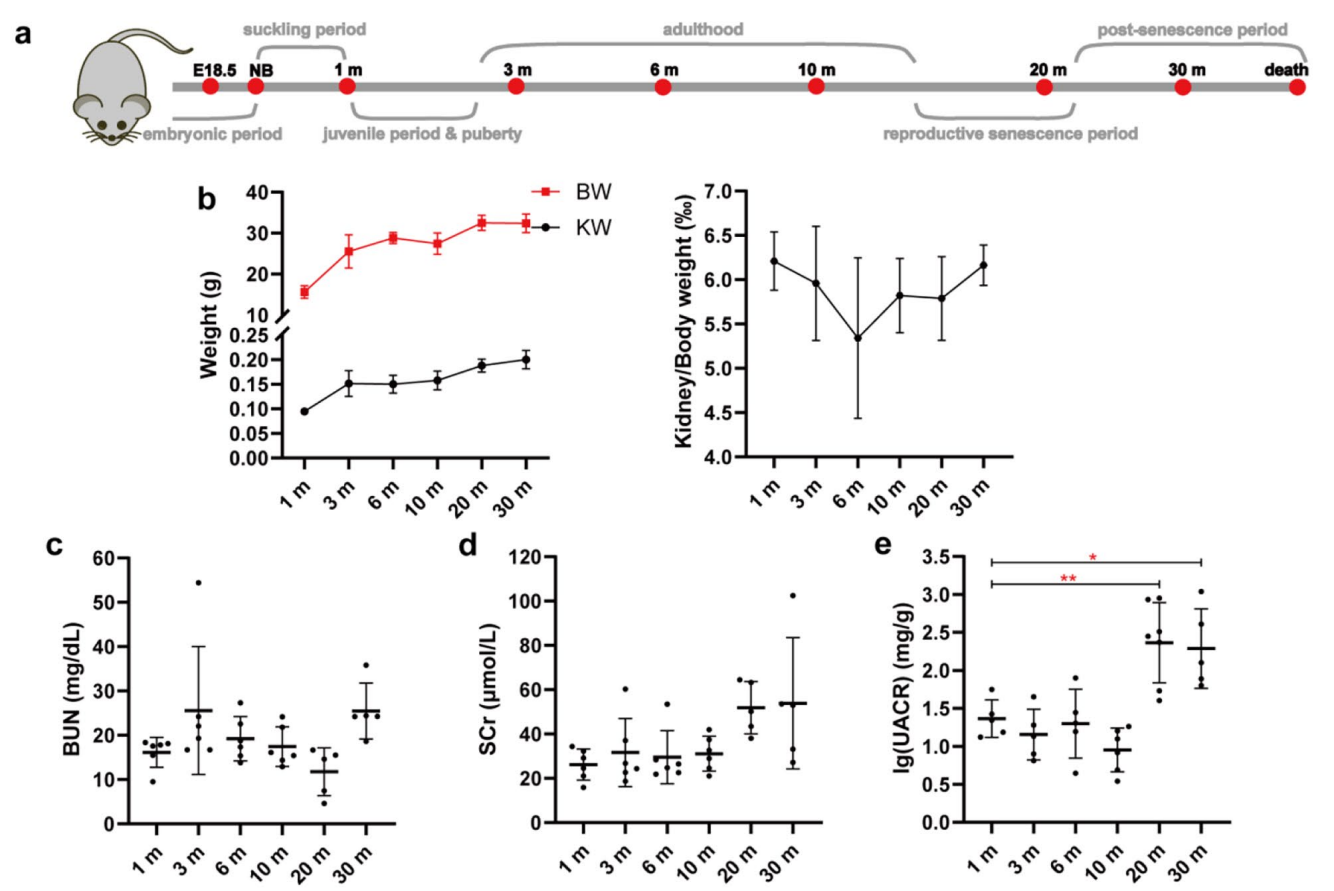
Biological parameters at various developmental stages of C57BL/6 mice. **(a)** The different life stages corresponding to the groups of mice studied. **(b)** The basic conditions including body weight (g), kidney weight (g), and kidney-to-body weight ratio (‰) of mice. **(c-e)** Serum creatinine (SCr), blood urea nitrogen (BUN) and urine albumin creatine ratio (UACR) levels in the eight groups. Data are expressed as the mean ± SD. *N* = 6 for each group. *P < 0.05, **P < 0.01, ***P < 0.001

We divided the mice into eight groups, with five to six mice each, and each group respectively represented embryonic stage (embryos 18.5 days), suckling period (1 day after birth), juvenile stage (1 month old), adulthood (containing 3 months old, 6 months old and 10 months old), reproductive senescence stage (20 months old), and post-senescence stage (30 months old) of mice (Fig. 1a). Significantly, the kidney volume of senescent mice was increased compared with maturing and middle-aged mice (Fig. 1b). Blood and urine samples were collected at the time of euthanasia. Biological parameters, including the serum creatinine (Scr), BUN, UACR were analyzed (Table 1). There were no significant changes in the level of Scr and BUN in different groups. Compared with the adulthood, the level of UACR had a slight increase in the senescent mice (Fig. 1c-e).

**Table 1:** Blood biochemical indicators of mice in the indicated groups

	1 m	3 m	6 m	10 m	20 m	30 m
LDL(mmol/L)	0.47±0.11	0.44±0.15	0.46±0.09	0.44±0.07	0.44±0.05	0.48±0.20
HDL(mmol/L)	1.05±0.15	1.20±0.26	1.28±0.19	1.08±0.08	0.90±0.21	0.92±0.36
TG(mmol/L)	0.43±0.12	0.55±0.23	0.57±0.28	0.41±0.19	0.44±0.12	1.05±0.67
CHOL(mmol/L)	2.53±0.47	2.70±0.57	2.84±0.21	2.52±0.16	2.07±0.46	2.05±0.60
ALT(U/L)	48.86±15.48	62.75±13.84	60.85±19.57	37.95±4.42	88.08±21.06	46.93±13.54
AST(U/L)	207.76±106.68	155.62±30.84	165.05±41.54	140.81±40.42	260.93±135.47	164.48±75.41

LDL, low density lipoprotein; HDL, high density lipoprotein; TG, total cholesterol; CHOL, cholesterol; ALT, alanine aminotransferase; AST, aspartate aminotransferase. 6 mice per group. Data are expressed as the mean ± SD.

### Kidney histopathology at various stages

**Fig. 2:**
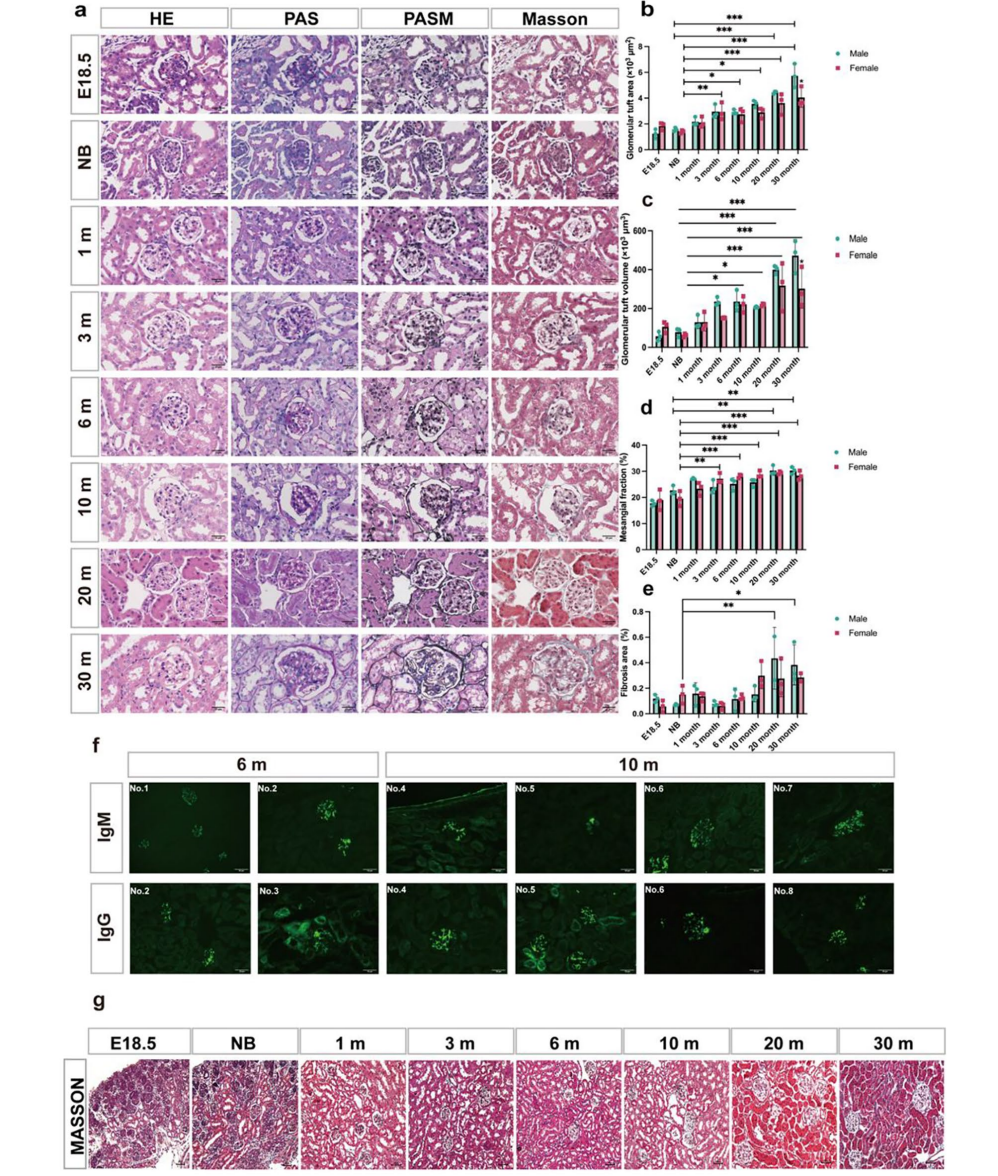
Renal histopathology at various developmental stages of C57BL/6 mice. **(a)** The four basic stains (HE, PAS, PASM, Masson) of kidney pathology in the indicated mouse groups. Scale bars, 25 μm **(b, c)** Glomerular tuft area and glomerular tuft volume of each group. *n* = 3 mice per group*7–9 glomerular per mice. **(d)** Quantification of the mesangial matrix fraction. *n* = 3 mice per group*4–6 glomerular per mice. **(e)** Quantification of the fibrosis area. *n* =  3 mice per group*4–6 glomerular per mice. **(f)** Immunofluorescence staining for IgG and IgM in kidney sections from different life stages of mice. Scale bar = 50 μm. Typical images are shown. Calculation of glomerular area and volume and quantification of mesangial expansion was based on glomeruli cut at the vascular pole per mouse in each group. Data are expressed as the mean ± SD. *P < 0.05, **P < 0.01, ***P < 0.001. **(g)** Representative Masson images

Compared with maturing and middle-aged mice, both male and female, there was a slight increase in the glomerular size and mesangial width with age as shown by the silver and PAS staining. (Figure 2a and d). No significant fibrosis was present in maturing and middle-aged mice, though total fibrotic area of the kidneys showed a mild increase from 20 month of age in male (Fig. 2e and g).

To clarify whether immune reactions were present in the developing C57BL/6 mice, we examined the deposition of immunoglobulin (Ig), IgG, C3, IgA, IgM, and C1q in glomeruli of all groups. The results demonstrate that by 6 months, deposition of IgG and IgM was present in glomerular mesangial areas and segmental capillary walls (Fig. 2f).

**Fig. 3:**
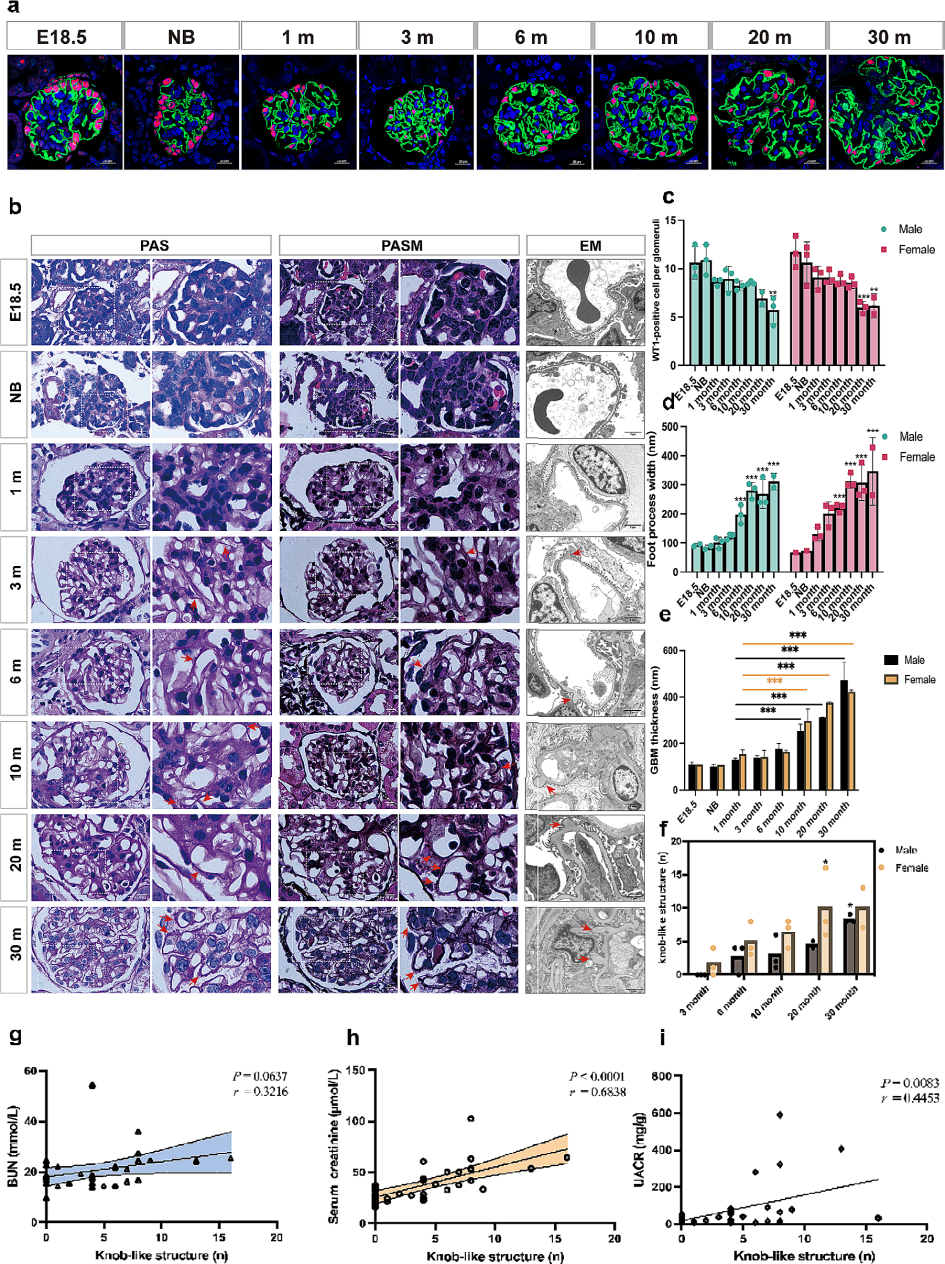
Podocyte characteristics and GBM changes with age in mice. **(a)** WT1 and synapotopodin in kidney sections from the indicated mouse groups. Scale bar = 20 μm. **(b)** Display of knobs-like structure under light microscope (PAS, PASM staining) and electron microscope. Scale bar of light microscope = 10 μm. Scale bar of electron microscope = 10 μm. **(c)** Quantification of WT1 + podocytes per glomerular cross-section. **(d)** Quantification of foot process width. **(e)** Quantification of GBM thickness. **(f)** Quantitative statistics and analysis of knobs-like structures in each group. **(g)** Correlation analysis between number of knobs-like structures and Scr. **(h)** Correlation analysis between number of knobs-like structures and Bun. **(i)** Correlation analysis between number of knobs-like structures and UACR. 3 mice per group. Data are expressed as the mean ± SD. *P < 0.05, **P < 0.01, ***P < 0.001

Podocyte number was stable from new-born till the age of 10 months. For female, by the age of 20 months, the number of WT1 positive podocytes was reduced till the age of 30 months, while this change does not occur until the age of 30 months in male mice (Fig. 3a and c). Significant GBM alterations were observed as presented by the increased thickness and knob-like structures beginning from 3 months of age till senescence at 30 months as detected by the silver staining, PAS staining and electron microscopy (Fig. 3b and d). With age, the number of knobs per 10 glomerular capillary walls (GCW) was significantly increased. Compared with the maturing and middle-aged mice, the thickness of GBM and the foot process width (FPW) was increased with age and the difference reached statistical significance at 6 and 10 months of age (Fig. 3d-f). In addition, the number of knobs was positively correlated with both UACR and Scr. (Fig. 3g-i)

### 10X Genomics single-cell transcriptome analysis of the kidney at various stages

**Fig. 4:**
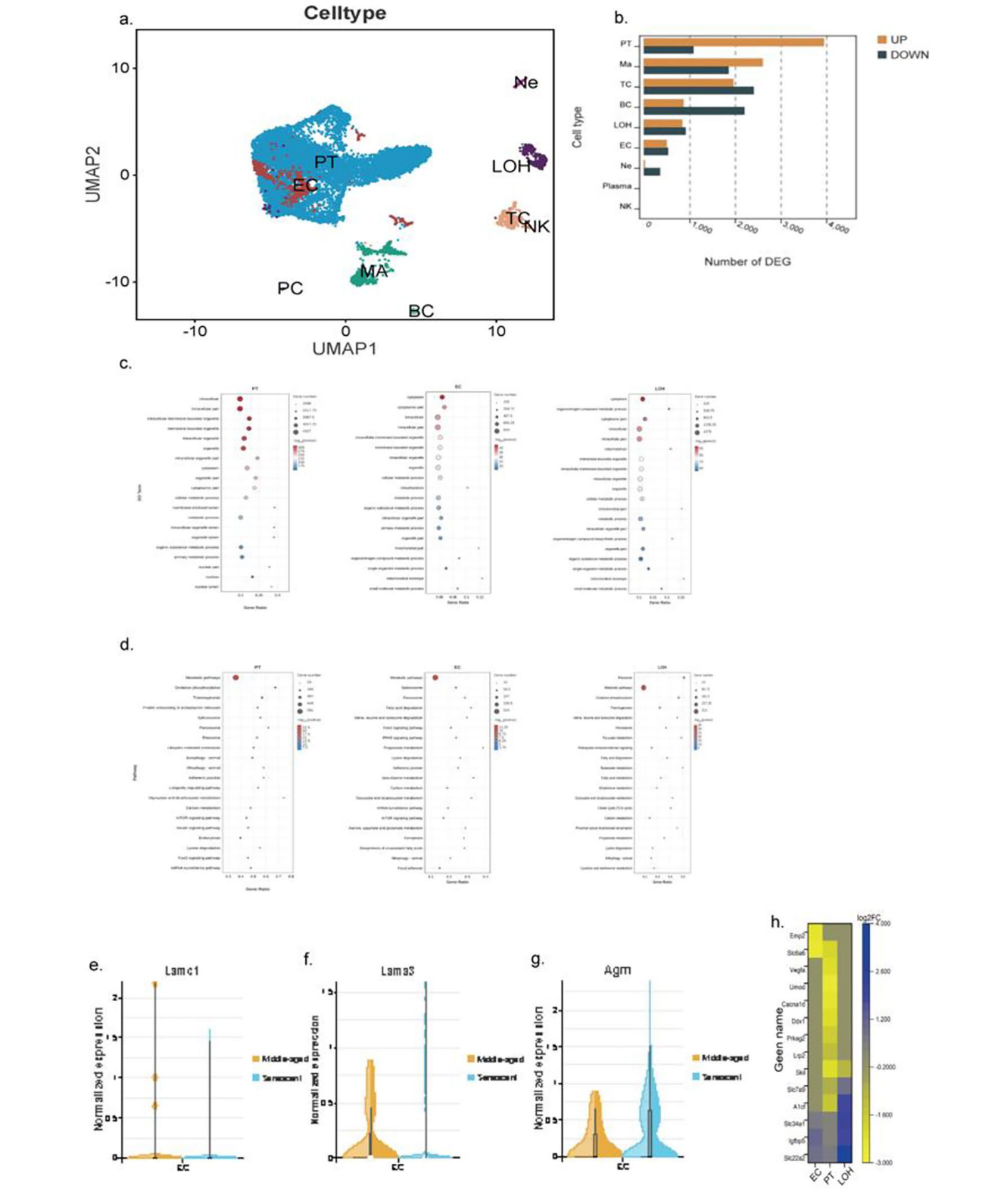
Single-cell transcriptome analysis reveals differences in gene levels with age in mice. **(a)** soft k-means clustering analysis of whole kidney cells identifies 14 cell clusters corresponding to 12 different cell types. **(b)** Differentially expressed genes in different cell types. **(c,d)** GO and KEGG enrichment analysis of genes within senescent mice compared with maturing mice. **(e,f,g)** Respectively represent the normalized expression of Lamc1, Lama3 and Agrn gene in middle-age and senescence. **(h)** Single cell-type specific average expression of human monogenic disease genes. Mean expression values of the genes were calculated in each cluster. In the heatmap, each row represents one gene and each column is single cell type

A total of 10, 895 and 4408 cells were isolated from whole kidney cell suspensions from the young and senescent mice, respectively. 10X genomics single-cell sequencing was conducted and stringent quality controls were performed using cellranger [12]. Clustering analysis using soft *k*-means clustering revealed 14 cell clusters and 7 different cell types. These included intrinsic glomerular cells and tubular cell types (Fig. 4a). Of note, proximal tubular epithelial cells (PTCs) had a few numbers of differentially expressed genes (DEGs) (Fig. 4b). KEGG enrichment for biologic processes (Fig. 4c) showed that various processes, including and focal adhesion, metabolic pathways, and signaling pathways (mTOR signaling) were enriched in cluster of glomerular cells and tubular cells. Furthermore, GO enrichment indicated that there was enrichment in processes involved in mitochondrion and metabolism (Fig. 4d). By 30 months, compared with maturing mice, typical characteristics of single-cell transcriptome analyses were shown as presented by differential expression of genes that encode the GBM’s components (Lamc1, Lama3, Agrn) in clusters of ECs (Fig. 4e-g). Further, we also explored whether aging-related phenotypes in the mouse kidney could be inferred from the expression pattern of human genes, whose loss of function results in kidney diseases. Of significance, we found that the mouse homologs of epithelial membrane protein 3 (EMP3), which have been associated with monogenic inheritance of proteinuria in humans were obviously upregulated in ECs. Furthermore, genes that have been implicated in chronic kidney disease (CKD), such as Slc6a6 were up-regulated in ECs, and Umod, Cacna1d, Ddx1 were specifically up-regulated in PTs (Fig. 4h).

## Discussion

This study reports a comprehensive atlas of the renal histopathology and single-cell transcriptome analysis at various developmental stages in the inbred mouse strain C57BL/6. Main findings are as follows: (1) compared with the maturing and middle-aged mice, the level of UACR had a slight increase in the senescent mice; (2) mesangial width gradually increases from 20 months of age; 2) with age, there were immune reactions involving IgG and IgM depositions in glomeruli of C57BL/6 mice by 6 months; 4) podocyte number remains stable from the young till 10 months of age, and was reduced till 30 months of age; 5) GBM is thickened and typical knob-like structures are present from 3 months and gradually increases with age; 6) single-cell transcriptome analysis reveals: compared with the maturing mice, the level of basement membrane component genes *Lamc1*, *Lama3*, *Agrn*, shows significant differences in the aging mice. These results offer the baseline reference values at various developmental stages of C57BL/6 mice under the physiological condition.

The most striking feature of the C57BL/6 mouse kidney is the characteristic nodules protruded on the subepithelial side of the GBM appearing from 3 months old till senescence at 30 months old. Similar GBM nodules have also been reported in several studies and researchers are arguing whether this alteration is age-related or gene deficiency related phenotype. In ddY mice, a murine model for spontaneous IgA nephropathy, there is a progressive increase in glomerular extracellular matrices, including the GBM thickening and formation of GBM nodules [16]. Of interest, studies have also shown that aminopeptidase A (*APA*) knockout mice exhibited a distinctive appearance of knob-like structures, supporting a role for *APA* in the maintenance of glomerular structures [17]. Other studies also report that in Agrin knockout mice, charge selectivity is a feature of GBM changes [18]. In the knockout mice of discoidin domain receptor 1 (*DDR1*) gene, the receptor tyrosine kinase for type IV collagen in basement membranes, electron microscopy showed a localized, subepithelial, mushroom-like isodense thickening of the GBM with a focal loss of the podocyte slit diaphragms. This study suggests that the interaction between type IV collagen and DDR1 plays an important role in maintaining the GBM integrity [19]. Though different terms are used in different studies, the main component of these GBM nodules is the cell matrix. In this study, we found that similar GBM knobs were present starting from 3 months of age and becoming frequent with age till 30 months. This result suggests that whether it is called GBM nodules, knob-like structures, or mushroom-like isodense GBM thickening, the appearance of this typical phenotype is age-related in mice. Noteworthy, gene deficiency could also cause the GBM bulges, the main difference lies in the occurring age, number, and size of the nodules. For instance, in 4-weeks-old *APA* knockout mice, the GBM knob-like structures are seen with the number increasing significantly at week 12 compared to age-matched wild type littermates [17]. These data indicate that when analyzing the GBM phenotype using C57BL/6 mice as an experimental model, the animal age should be cautiously selected.

Mice are now recognized as the preferred experimental model for numerous preclinical areas pertaining to human diseases, including diabetes research, on account of their comparatively economical nature, strong procreation, abbreviated gestation period, and straightforward genetic manipulation capabilities. In both human and experimental animals, males have renal failure at a quicker rate than females [20]. The animal gender may be another influencing factor in different individual studies. As a result, our study provides a range of sex-related phenotypic differences for reference. Kidney functions are determined by sex, androgens induced kidney overgrowth in developing mice by inducing an initial surge of cell proliferation followed by a subsequent enlargement of cell size [21]. Of course, in individual experimental settings, gender should be carefully considered.

The GBM, with podocytes and glomerular endothelial cells on either side, comprises the critical glomerular filtration barrier and functions as both a size and charge selective filter. It is a mixture of extracellular matrix composed of various substances, including collagen IV, laminin, and heparin sulfate glycosaminoglycan, etc. [22, 23]. Genes in both podocytes and endothelial cells may regulate the secretion of GBM components and their balance plays a critical role in maintaining its physiological function. To get a deep insight into the mechanisms of this specific GBM knob-like phenotype, we applied single-cell transcriptome analysis to explore the profiles of GBM-associated genes and potential interactions between glomerular cells. Laminin, type IV collagen, nidogen, and heparan sulfate proteoglycan are the four main macromolecules that make up GBM, an extracellular matrix that resembles a sheet. The major one in GBM is agrin [24]. Laminin is necessary for the development of a basement membrane in both the early embryo and the embryoid body [25]. The lack of the γ1 chain results in distinct processing of the remaining subunits within the trimer, hence impeding the formation of a laminin molecule capable of polymerisation [26]. In mice lacking the *LAMC1* gene (laminin γ1 gene), the basement membrane did not form, and only irregular deposits of collagen IV were found beneath the epithelia [27]. LN α3 appears to be required for glomerular endothelial cells (GEnCs) maturation. In *Lama3*^*−/−*^ mice, endothelial cells migrate into the GBM scaffold deposited by podocytes. GEnCs generate their own basement membrane and adhere to it, although they are unable to successfully generate fully functional tubular structures or fenestrations [28]. Agrin is the major heparan sulfate proteoglycan of the GBM [29], dramatic reduction in the GBM anionic charge was detected when agrin was removed from the GBM by podocyte-selective knockout of Agrn. Our results have shown that genes of laminin and agrin expression were significantly upregulated in endothelial cells. These increased components could be the result of this GBM phenotype. However, in human kidneys, such GBM nodules were not seen at the old age, nor were reported in the literature [30]. This suggests that the GBM knob-like features may be species-specific. In C57BL/6 mice, a robust murine model used for pathogenesis study, enough attention should be paid when the GBM was analyzed, especially in diabetic nephropathy when the GBM thickness is a gold standard for evaluating the disease state. Remarkably, proper age-matched controls should be set in designing the individual experiment. This partly explains why in most studies, the age of mice is usually between 6 and 8 weeks when in this period the histopathology and transcriptomics are regarded as biologically normal.

Mice and human share a significant portion of their genetic makeup, which allows mice to serve as useful models for many human diseases and biological processes. The biological and pathological profiles of the kidney would be a novel and crucial resource for mechanistic insights and testing of potential therapeutic interventions. Noteworthy, cross-species comparisons on the transcriptomes of the principal glomerular cell types validated consistent differential expression pattern between the two species for most of the genes [31]. Hence, further validation is required on the specific molecular mechanisms of the formation of GBM knob-like phenotype in our study.

Our kidney cell atlas also clarifies the pathogenesis of aging and kidney disease. We discovered that the mouse homologs of the human genes causing kidney disease to have been associated with aging. From this perspective, it may be argued that age plays a distinct role in the identification of potentially influential genes associated with kidney disease.

The current data provides the fundamental reference values for renal histopathology and biological parameters during different developmental stages of C57BL/6 mice under normal physiological conditions. It is noteworthy that the GBM knob-like structures, which are observed in C57BL/6 mice, are associated with senescence, and emerge at 3 months of age. Various stages of development have distinct phenotypes that may affect the interpretation of the results. Consequently, careful consideration should be taken when selecting the age of C57BL/6 mice for individual settings.

## Data Availability

All data underlying the findings reported in this manuscript are provided as part of the article. The proteomics data are available via GEO with identifier GSE262959.

## Declarations

### Summary statement

The availability of comprehensive kidney morphology and single-cell transcriptome atlas at various developmental stages of C57BL/6 mice.

## Abbreviations

GBM: Glomerular basement membrane
scRNA-seq: Single-cell sequencing
ECs: Endothelial cells
BW: Body weight
BUN: Blood urea nitrogen
Scr: Serum creatinine
UACR: Urine albumin to creatinine ratio
KW: Kidney weight
H&E: Hematoxylin and eosin
PAS: Periodic acid-Shiff
PASM: Periodic acid-silver methenamine
MTS: Masson’s trichrome staining
GEMs: GelBeads-In-Emulsions
SD: Standard deviation
Ig: Immunoglobulin
WT1: Wilm’s tumor 1
GCW: Glomerular capillary walls
FPW: Foot process width
PTCs: Proximal tubular epithelial cells
DEGs: Differentially expressed gens
CKD: Chronic kidney disease
EMP3: Epithelial membrane protein 3
APA: Aminopeptidase A
DDR1: Discoidin domain receptor 1
GEnCs: Glomerular endothelial cells
